# Indents

**Published:** 1914-07-15

**Authors:** 


### INDENTS.

☞Brief Articles of Technical or Scientific Value will receive notice and comment. Contributions invited.

*How it was done in Olden Times.* When I began dental practice upper dentures were held in place by spiral springs which curved around in the cheek, attached to the denture between the molars and bicuspids. In case it was only an upper denture, the attachment was made to the lower jaw with a gold band on a tooth. Plates did not cover the palate as it was not necessary. In London fourteen years ago, I was surprised to find dentists still using this method.

In 1844, my preceptor conceived the idea of securing the plate in place by suction. In order to do this more successfully, he thought an impression of plaster would be preferable to the beeswax then used universally. Using the common plaster of commerce he secured an impression, and proceeded to construct a denture on gold for an old man.

His die metal was tin, but as lead for a counter die could not be poured on the tin he cast the counter die first by holding the plaster model, very dry, in the lead, then placing a rim of several thicknesses of paper around the counter poured the tin. Later on he used zinc for dies.

Upon swaging the plate and trying in the mouth he was surprised to see how it adhered In order to make a test he soldered a loop to the center of the plate, attached a long iron wire, placed it in the mouth, and told the patient to suck it up, then hung a large pail full of water to it, and the pail was suspended. Of course no further use for spiral springs.

It will be observed that the plate came in contact with the membrane all over the jaw. Some years later a dentist named Gilbert, thought to improve the adhesion by means of an air-chamber, and it came into general use, known as the "Gilbert air-chamber."

The original principle of close contact with the membrane is used now, only a "relief" is applied over the hard palate, to prevent the plate resting there and rocking as change takes place, sooner or later on the ridge.—Dr. L. P. Haskell in *Review.*

*How to hold Cotton on a Smooth Broach.* There is one point in technique with reference to the treatment of pulp canals which I learned on a trip to New Orleans, a point I consider well worth the expense of the trip a great many times over. It had been my custom to dry pulp canals by twisting cotton fiber upon a spiral broach. Occasionally it was almost impossible to get the cotton off of the broach, consequently I lost much time. I tried many times to use a smooth broach as an applicator for medicaments, by twisting cotton upon the broach, but I could not get it to hold. For instance, when I wished to apply a drug in the canals and pump it down towards the ends of the roots the cotton would become loosened. It is an easy matter to attach cotton to a smooth broach and all there is to it is to draw the smooth broach across a piece of sterile beeswax before twisting the fiber upon the broach. Any amount of cotton may be firmly attached to the broach and it will not become loosened in pumping the drug into the canals. That little point in technique has saved many hours of time for me. It has materially assisted me in drying canals because I am enabled to use a smooth broach of extremely small diameter and twist a few, or as many fibers as necessary, upon it, and come more nearly approaching the apex of the roots with the dry cotton fibers in drying the canals than by any other method.—Carl D. Lucas in *Dental Review.*

*Teeth and Health.* In a recent number of the *American Journal of the Medical Sciences,* Camac called attention to the importance of a more intimate co-operation between the physician and the dentist. There is little doubt that for many years the medical profession has assumed a somewhat superior position, and has refused to recognize the bearing that dental disturbances have upon the general health.

Anyone who takes the trouble to examine, even casually, the oral cavities of his patients will be astonished at the condition of the teeth. Although American soldiers have excited favorable comment on account of the tooth-brush being so much in evidence, yet the care of the mouth is neglected sadly by the community. So many teeth are gone that the proper mastication of food becomes impossible, and the individual is commonly underweight and anemic. In addition to this loss of mechanical value, there is the presence of the necrotic conditions that led to the destruction of the teeth. It may be called Riggs' disease, pyorrhea alveolaris, dental abscess, dental sepsis, etc., but the important point in common is the presence of pus in greater or lesser amount. If, as is not infrequent, two or three drams of pus, containing virulent streptococci, are secreted and swallowed every day, there must occur some disturbance of digestion. But there is an even more serious and dangerous possibility, that of localized collections of pus which cannot escape, and may therefore give rise to systemic infections, such as arthritis and endocarditis. Such a purulent condition anywhere else than around the teeth would be attacked most vigorously. No physician would permit an infected finger-nail to go untreated, yet the same man will calmly disregard a suppurating tooth and wonder why his treatment is not successful. Camac believes that consultations of the internist with the dentist are as necessary as those with the surgeon, a belief that is indeed warranted by the facts.

The foregoing free confession of delinquency on the part of physicians in relation to serious consideration of oral condition, and unqualified recognition of the vital importanc of the dental factor in disease, is most encouraging to that dental practitioner who has learned to look beyond the merely technical part of his work.—Editorial in *New York Medical Journal.*

*Unreliability of Mouth-Washes.* A pathetic figure in our profession is the man who relies implicitly upon the therapeutic values of various mouth-washes and remedies to alleviate certain pathological conditions in the mouth. Does this sound familiar to anyone? "Doctor, when I brush my teeth, my gums bleed. Is there anything I can use?" "Why certainly," promptly responds the doctor. "Get a bottle of Blank's Germicidal Astringent Deodorizing Tissue-building Mouth Reviver, and use it every morning." "Will that stop the bleeding," asks the patient? "Why, certainly! If it does not, massage the gums vigorously after each meal."

While I do not believe that this practitioner has been induced by a generous supply of samples to campaign for a new proprietary mouth-wash, yet he has failed to diagnose the cause of the congested condition of the gums. He does not realize that he is prescribing a mildly antiseptic solution to perform the miracle of dislodging flint-like calcarious deposits that are impervious to its action, and are only amenable to surgical removal. To prove still further that he does not know what is wrong, he advises a vigorous massage of the soft gum tissue over these flint-like nodules, which cannot in any conceivable manner act in any other way than as an added mechanical irritation to the tissues. In some conditions mouth-washes are indicated, but all the mouth-washes in the country would not have sufficient remedial value in cases similar to these. In some cases massaging the gums is highly beneficial in stimulating the tissues, but the indiscriminate manner of prescribing mouth-washes and massaging as a panacea, as is often done, shows a disgraceful lack of thought. The deposits on the teeth are as foreign to their surroundings as would be a splinter in one's finger. Imagine the value of a lotion and massage for a swollen finger!

The worst of all mercenary mountebanks who filch from as unsuspecting public is he who, shielded by a diploma of Doctor of Dental Surgery, knowingly shirks evidently needed prophylactic operations and does other work solely because he thinks he can procure a higher fee. He may be apt enough to realize the value of restoring the mouth to an hygienic condition, but it is the almighty dollar that he is after, and he will acquire that dollar in the quickest manner his sordid mind can devise.—Dr. Gearhart in June *Cosmos.*

*Nitrous Oxid-Oxygen Anesthesia.* The use of oxygen in conjunction with nitrous oxid for producing *narcosis* is a delusion. It is the stumbling block that causes a great many failures. Our literature is full of the percentages of oxygen to give and I want to say, that from some experience at least I have use for no cylinder oxygen. This might not be true if we had a perfect machine. The defects in inhalers and valves have given our patients enough oxygen in most cases to do away with the necessity of cylinder oxygen turned into a bag expressly for the purpose of giving percentages. I'll grant that if you enclose your patient so that no oxygen can get to his lungs you will soon have him in a dangerous condition; but you don't so enclose him and consequently he gets atmospheric oxygen sufficient to the anesthesia demanded in short operations. If the prolonged administration be essayed you should give oxygen either from cylinder or air in such proportions as will meet the exigencies of the case. To lay down a rule of percentages is simply assuming that there is something about patients mathematically correct and exact and undeviating, an assumption at once preposterous and ridiculous. My advice would be—spare the oxygen, at least in the early stages. Further in regard to the subject of oxygen in nitrous oxid anesthesia—the type of patient must be considered before starting the administration. Use no oxygen in the early stages of anesthesia with the vigorous, high lighted type represented very concretely in certain Irish and French. In anemic people you must use oxygen in increasing proportions as the period is prolonged.

In a great many oral operations, particularly the extraction of teeth where more or less roughness is used in consequence of the resistance of the tooth to removal, the anesthesia is very apt to be interfered with by what are called inter-asphyxial symptoms. Oxygen from cylinder or air is here indicated until the convulsions and syanosis have disappeared when the nitrous oxid may again be administered. During such periods of interference it is quite necessary that the blood be sponged from the mouth and that the patient be kept in a position bolt upright, thus preventing blood from lodging in the pharynx and making the conditions worse by absolutely stopping the ingress of air to the lungs. The operations of extraction should be performed with as much gentleness as possible to avoid these symptoms. And I must add a caution right here, concerning the mouth prop. On account of a lack of muscular relaxation, no one should presume to administer a nitrous oxid anesthetic without having a mouth prop in position from the beginning. In an unrelaxed condition, it might be necessary to have access to the mouth to draw forward the tongue in case of respiratory embarrassment.—Dr. Stowell in June *Review.*

*Typhoid Death Rate in the Army.* The death rate from typhoid fever in the U. S. Army has been reduced from 16.5% for 100,000 people in 1912 to no deaths in the 100,000 officers and men who were protected by vaccination during the past year. This showing makes the soldier safer than the civilian so far as this particular disease is concerned. If we could only discover a vaccine that was equally effective for tuberculosis, cancer, rheumatism, etc., we should be a happier people. Let us hope science may yet come to our rescue.

*Porcelain Restoration.* We frequently see anterior teeth in which the recession of the gums is so great that the exposed roots are unsighly, although there is no caries, and the teeth are apparently firm. In some such cases I have prepared cavities in the denuded roots, and, instead of using properly shaded porcelain as it comes to us, I selected the white porcelain as a foundation and finished up with what is known as "original gum enamel" used in continuous-gum work. In this way the appearance and the original shape of such teeth can be beautifully restored. This gum enamel is most useful in crown and bridge restorations, when, for instance, the right central incisor or the left lateral has been lost for a number of years and the absorption of the gum is so great that if a substitute were to be inserted so as to reach to the gum line, the substituted tooth would have to be much longer than the original natural tooth that the resulting appearance would be most unsatisfactory to dentist and patient alike. In order to produce a sightly and artistic restoration in such cases, I select a facing of proper length, mark on it the length of the corresponding natural tooth, grind off the enamel extending beyond this line of demarcation to the natural gum, and on this ground surface bake gum enamel of proper shade. —Dr. A. P. Burkhart in *Dental Cosmos*.

*The Removal of Compound From Cast.* When from overheating it seems almost impossible to remove compound, do not cut and ruin the cast with a knife, but in the usual way press the warm mass of compound against the clinging parts and hold for a second or two, and if it does not come away dip the cast in cold water once or twice, keeping mass of hot compound ready and it will easily come off after the cast is chilled a little. We find this method much better than the chloroform solvent and quicker, as it cannot penetrate the cast.—Walter A. Loope, D. D. S., Cleveland, Ohio.

*Aid in Taking an Impression for a Bridge.* Cases often present in which difficulty is encountered in taking an impression for a bridge. If milk of magnesia is painted over the parts to be reproduced, the impression may be taken out without any discomfort to the patient or trouble to the operator.—L. Kohn, *Odontologist*.

